# A Personalized Automated Messaging System to Improve Adherence to Prostate Cancer Screening: Research Protocol

**DOI:** 10.2196/resprot.2398

**Published:** 2012-11-28

**Authors:** Michael Juntao Yuan, Emily T Hébert, Ron K Johnson, Ju Long, Elizabeth A Vandewater, Andrew J Vickers

**Affiliations:** 1 Ringful Health, LLCAustin, TXUnited States; 2 University of Texas School of Public HealthDivision of Health Promotion and Behavioral SciencesAustin Regional CampusAustin, TXUnited States; 3 Texas State UniversityDepartment of Computer Information Systems & Quantitative MethodsSan Marcos, TXUnited States; 4 Michael & Susan Dell Center for Healthy LivingAustin, TXUnited States; 5 Memorial Sloan-Kettering Cancer CenterDepartment of Epidemiology and BiostatisticsNew York, NYUnited States

**Keywords:** Early Detection of Cancer, Text Messaging, Prostatic Neoplasms

## Abstract

**Background:**

Public adherence to cancer screening guidelines is poor. Patient confusion over multiple recommendations and modalities for cancer screening has been found to be a major barrier to screening adherence. Such problems will only increase as screening guidelines and timetables become individualized.

**Objective:**

We propose to increase compliance with cancer screening through two-way rich media mobile messaging based on personalized risk assessment.

**Methods:**

We propose to develop and test a product that will store algorithms required to personalize cancer screening in a central database managed by a rule-based workflow engine, and implemented via messaging to the patient’s mobile phone. We will conduct a randomized controlled trial focusing on prostate cancer screening to study the hypothesis that mobile reminders improve adherence to screening guidelines. We will also explore a secondary hypothesis that patients who reply to the messaging reminders are more engaged and at lower risk of non-adherence. We will conduct a randomized controlled trial in a sample of males between 40 and 75 years (eligible for prostate cancer screening) who are willing to receive text messages, email, or automated voice messages. Participants will be recruited from a primary care clinic and asked to schedule prostate cancer screening at the clinic within the next 3 weeks. The intervention group will receive reminders and confirmation communications for making an appointment, keeping the appointment, and reporting the test results back to the investigators. Three outcomes will be evaluated: (1) the proportion of participants who make an appointment with a physician following a mobile message reminder, (2) the proportion of participants who keep the appointment, and (3) the proportion of participants who report the results of the screening (via text or Web).

**Results:**

This is an ongoing project, supported by by a small business commercialization grant from the National Center for Advancing Translational Sciences of the National Institutes of Health.

**Conclusions:**

We believe that the use of centralized databases and text messaging could improve adherence with screening guidelines. Furthermore, we anticipate this method of increasing patient engagement could be applied to a broad range of health issues, both inside and outside of the context of cancer. This project will be an important first step in determining the feasibility of personalized text messaging to improve long-term adherence to screening recommendations.

## Introduction

Cancer is the second most common cause of death in the United States. In 2012, it is estimated that 1.6 million new cancer cases will be diagnosed and 577,190 Americans will die from cancer [1]. At least half of all new cancer cases can be prevented or detected by screening. Cancer screening not only prevents unnecessary cancer deaths, but it also can reduce the morbidity of cancer before it progresses to more advanced aggressive stages [2]. Despite the benefits of cancer screening, public adherence to guidelines for cancer screening is often poor. *Healthy People 2020*, the 10-year health agenda released by the US Department of Health and Human Services, reported only modest screening rates for breast cancer, cervical cancer, and colorectal cancer. For example, only 59% of adults reported being up-to-date with colorectal cancer screening [3].

Although screening can be beneficial, it is important that patients consult their doctor to determine which types of screening are appropriate for them. Screening guidelines vary widely depending on the type of cancer, patient age, gender, ethnicity, and family history [2]. Patient confusion over multiple recommendations and modalities for cancer screening has been found to be a major barrier to screening adherence [4]. Complicating this matter is the fact that cancer screening schedules often need to be dynamically adjusted for each individual based on previous screening results and comorbidities. For example, the recommended interval between prostate-specific antigen (PSA) tests depends on the most recent PSA level, with longer intervals (ie, less frequent testing) for men with lower PSA levels [5]. Similar dynamic screening and intervention algorithms have been suggested for other types of cancers, such as breast cancer [6,7].

Patient engagement and reminders improve adherence to cancer screening guidelines [8]. The Community Preventive Services Task Force, an independent group of public health experts appointed by the Director of the Centers for Disease Control and Prevention, recommends using client reminders to increase cancer screening based on strong evidence of effectiveness; reminders have been shown to increase mammography screening by a median of 10% and increase colon cancer screening by a median of 15% [9]. Although short message service (SMS) text-based reminders sent to mobile phones have not been studied specifically for cancer screening, SMS-based reminders have been demonstrated to reduce patient appointment no-shows by up to 40% [10], and reduce nonadherence with chronic disease follow-ups by up to 35% [11]. SMS reminders have been used successfully in driving positive behavior changes in areas such as medication compliance [12], weight loss [13], smoking cessation [14], and physical activity promotion [15]. A recent review of the use of SMS technology for health behavior change interventions found that SMS interventions yielded positive behavior changes in 93% of studies reviewed. The authors found that SMS was an effective delivery method for tailored advice and reminder messages for behaviors such as smoking cessation, diabetes self-management, and medication compliance [16].

In the United States, mobile messaging solutions have the potential to reach vast numbers of patients. It is estimated that 82% of American adults have cell phones, with the penetration rate skewed higher among African American and Latino populations [17]. A survey conducted in 2010 concluded that 79% of Medicaid patients with mobile phones are active SMS users [18]. Although this project focuses on prostate cancer screening, the product we propose to develop and test has wide-ranging implications for adherence to screening for many other types of cancers and for chronic disease management.

In this project, we propose to develop technology-based interventions, specifically, automated reminders sent to consumer mobile phones to help patients stay motivated and adherent with their personalized cancer screening schedules. The interventions are based on the theory of planned behavior [19], in particular the unified theory of acceptance and use of technology (UTAUT) [20] and the Patient Activation Measure (PAM) [21]. The UTAUT is a theoretical model and instrument used to assess the likelihood of user acceptance for new technology that posits that performance expectancy, effort expectancy, social influence, and facilitating conditions are direct determinants of use [20]. The UTAUT instrument is widely used to evaluate the factors affecting adoption of new technology solutions [22]. Patient activation refers to a person’s ability and willingness to manage their health and health care [23]. The PAM is a 22-item assessment used to evaluate an individual’s knowledge, beliefs, and confidence in managing their health [21]. High patient activation has been shown to be positively associated with utilization of preventive services, self-management behaviors, medication adherence, self-reported quality of care, and perceived doctor-patient communication [24-26].

We will conduct a randomized controlled trial to study the hypothesis that mobile reminders improve adherence to screening guidelines. Mobile messaging is an intervention that can be applied to a large population at low cost because it can be easily automated. We will also explore a secondary hypothesis that patients who reply to the messaging reminders are more engaged and at lower risk of nonadherence. The impact is that low-cost mobile messaging could be used as a signaling mechanism to predict patients with high risk of nonadherence, and resources could be optimized to target those patients for more intensive follow-ups.

In this project, we focus on prostate cancer to examine proof of principle for our general approach. We will use screening algorithms adapted from evidence-based prostate screening guidelines and multimedia content tailored for mobile phone use. We plan to subsequently develop and test algorithms for other types of common cancers, such as breast cancer and colorectal cancer.

## Methods

### Study Objectives

Our primary hypothesis is that use of the mobile messaging reminders will increase patient adherence to screening. To evaluate this hypothesis, we will conduct a randomized controlled trial in a sample of males between 40 and 75 years (eligible for prostate cancer screening) who are willing to receive text messages, email, or automated voice messages. The sample will be recruited from male clinic patients who may or may not be in the clinic for prostate-related problems at the time of recruitment.

### Eligibility Criteria

The project will solicit participation from men aged 40-75 years of all ethnicities who visit 3 designated primary care clinics or 2 area community health fairs in Texarkana and New Boston, Texas, and Hope, Arkansas. No one will be excluded from the study on the basis of race/ethnicity or disability. Participants must have Internet access at home or at work and be willing to receive message-based communications on their choice of communication channels, including mobile messaging, email, and automated voice messages. Participants must be willing to pay for any communication costs charged by their wireless carrier, Internet provider, or phone company. Participants will be excluded if they have been diagnosed with prostate cancer, have had a PSA test in the previous 12 months, or have a current appointment for prostate cancer screening.

### Recruitment

All eligible participants will be given a study information sheet upon check-in at the clinic. The information sheet will include a brief description of the study, including the study purpose, procedure, and relevant risks and benefits. The information sheet will instruct interested participants how to enroll in the study through a secure Web portal where they will give their informed consent and mobile phone number to the research team. When available, a trained research nurse will explain the details of the study to eligible participants, answer any questions, and give the participants the opportunity to enroll during check-in at the clinic. Depending on their communication preference stated at the time of sign-up, participants will receive a text message or email on their phone or a voice message asking them to confirm that they would like to participate in the study. Patients must respond yes to this message to enroll in the study.

### Study Procedures

After initial recruitment, participants will be asked to complete a brief questionnaire about their basic sociodemographic details and relevant health history, as well as a Technology Acceptance Model questionnaire [22] and the PAM [21] to measure patient engagement in health care. Participants will be randomly assigned to the intervention or the control group using a random number generator. Randomization will be implemented automatically by a secure password-protected algorithm at the time the user signs up. This guarantees that assignment cannot be predicted before or changed after the participant is registered in the study. All participants will be asked to schedule prostate cancer screening at the clinic within the next 3 weeks, but only the intervention group will receive reminders and confirmation communications for (1) making an appointment, (2) keeping the appointment, and (3) reporting test results back to the investigators.

**Figure 1:**
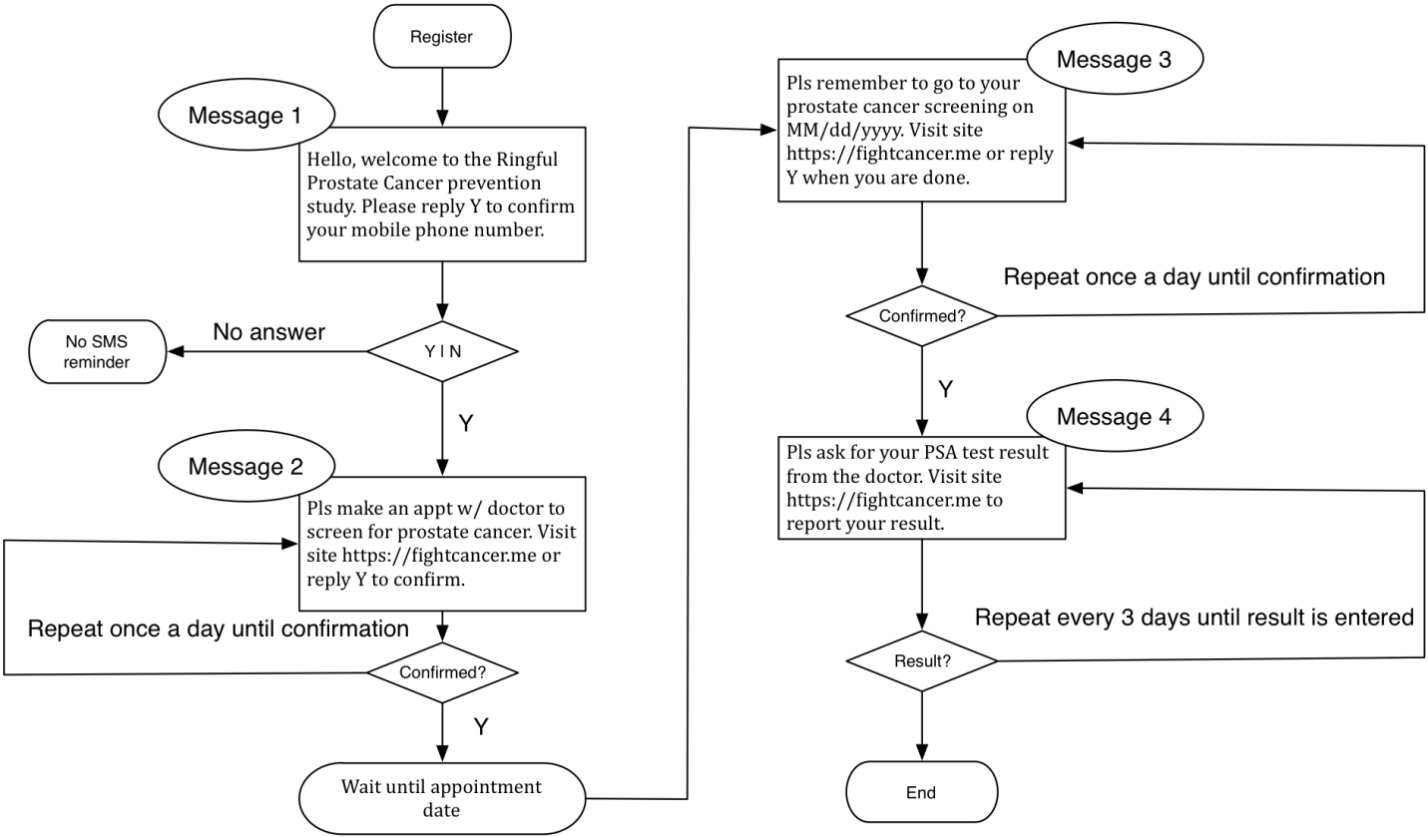
Study flowchart.

A flowchart of the reminders/confirmations and the expected responses from participants is illustrated in Figure 1.

### Screening Reminders

A screening schedule reminder will be sent to remind the participant to make an appointment for the screening test. One week after the participant confirms his appointment, the system will ask the participant whether he has seen the doctor yet. This message will repeat every 2 weeks until the participant confirms that he has kept the appointment and seen the physician. Then the system will ask the participant whether he has received his test results. This message will repeat every 3 days until the participant confirms that he has received the test results. The participant will then be asked to report back his test results to the research team using the secure Web portal.

In addition to the messages, we will also provide patient education and health promotion materials to the participants as part of the communication process. Upon initial registration on the secure website, a short video will explain the importance of prostate cancer screening and what to expect from the screening tests to both the control and intervention group participants. Once the intervention group participants confirm and report back their test results, another short video will explain ranges and meanings of test results, and the preventive actions the participant can take to reduce prostate cancer risk.

At the end of the study period, all patients will be contacted again through their preferred communication channels as indicated at sign-up (eg, text message, email, or voice message). All participants will be asked to report whether they have made appointments for prostate screening, kept their appointments, and reported back the test results. All participants will also be asked to complete a second questionnaire on the secure website that is a repeat of all initial questionnaire items except the sociodemographic items. The study period will last 8 weeks. Participants will receive a US $10 gift card as compensation for their participation in this study.

### Evaluation

Three outcomes will be evaluated: (1) the proportion of participants who make an appointment with a physician following a mobile message reminder, (2) the proportion of participants who keep the appointment, and (3) the proportion of participants who report the results of the screening (via text or Web). To facilitate comparison with existing literature, each outcome will be examined independently as a separate outcome. Adherence rates in the intervention and control group will be compared using the Fisher exact test.

Our second goal is to examine user adoption, identify factors that foster or hinder adoption, and identify patients who are at high risk of nonadherence. Examination of these questions will utilize data from the intervention group only. We hypothesize that those who respond to texts will show higher levels of adherence and patient activation than those who do not respond. For these analyses, we will focus on both user responses to reminders and adherence, which will be treated as a continuous variable by counting the total number of adherence behaviors (eg, made the appointment, kept the appointment, and followed up after results). Factors that foster or hinder adoption will be examined both preintervention and postintervention utilizing all available measures, particularly those related to technology acceptance factors defined in the UTAUT model. Examination of user adoption and factors fostering or hindering adoption will be accomplished through ordinary least-squares regressions. The UTAUT survey questionnaires are designed to measure 4 latent factors that drive technology adoption: performance expectancy, effort expectancy, social influence, and facilitating conditions. The first 3 factors are linked to behavior intention, another latent factor. We will conduct confirmatory analysis using structural equation modeling technique against the data. This will result in a path diagram that weights each latent factor’s contribution to user adoption of this technology. The analysis will help to identify the key drivers of user adoption and give the team directions for further product improvements.

The PAM survey contains 13 questions that measure 4 different aspects of patient activation. We will calculate a mean score and standard deviation for each question in the following groups: intervention, control, intervention who reported outcomes, and intervention who did not report outcomes. We will then detect any significant differences in PAM scores among those groups. A significant difference between intervention and control could indicate that the reminders themselves help activate the patient; a significant difference between the ones who reported outcomes and the ones who do not could provide a signaling mechanism to identify high-risk patients through automated means.

Based upon the patterns of adherence among patients receiving the intervention, it may be possible to identify those patients at highest risk for nonadherence and to identify which variables differentiate those at risk of nonadherence from those who are most likely to adhere.

## Discussion

The increasing complexity of cancer screening algorithms is such that the burden of ensuring compliance needs to shift from individual patients and health care providers toward centralized and automated systems. What may seem overwhelming for a patient or physician, such as graduated screening intensities contingent on the previous PSA level, can be implemented in a relatively straightforward fashion by computer. We believe that use of centralized databases and SMS text messaging could improve adherence with screening guidelines. Furthermore, we anticipate this method of increasing patient engagement could be applied to a broad range of health issues, both inside and outside of the context of cancer. This project will be an important first step in determining the feasibility of personalized text messaging to improve long-term adherence to screening recommendations.

## Multimedia Appendix 1

Reviewer comments and summary statement from NIH peer reviewers.

## Multimedia Appendix 2

NIH Approval and Funding.

## Multimedia Appendix 3

IRB Review and Approval.

## Abbreviations

PAM: Patient Activation Measure
PSA: prostate-specific antigen
SMS: short message service
UTAUT: unified theory of acceptance and use of technology
