# Supplementary material for: A Personalized Automated Messaging System to Improve Adherence to Prostate Cancer Screening: Research Protocol
Source: JMIR Res Protoc. 2012 Nov 28;1(2):e20. doi: 10.2196/resprot.2398 (PMC3626152; doi:10.2196/resprot.2398)
Supplement: Supplementary file 1 [file resprot_v1i2e20_app1.pdf]

SUMMARY STATEMENT
(Privileged Communication)

PROGRAM CONTACT:
David Beylin
301 496-0079
beylind@mail.nih.gov

Release Date: 07/15/2011

Application Number: 1 R43 CA162953-01A1

Principal Investigator

YUAN, JUNTAO MICHAEL

Applicant Organization: RINGFUL, LLC

Review Group: ZRG1 HDM-K (10)
Center for Scientific Review Special Emphasis Panel
Small Business: Healthcare Delivery and Methodologies

Meeting Date: 06/23/2011
Council: OCT 2011
Requested Start: 11/01/2011

RFA/PA: PA11-096
PCC: Z5BD
Dual IC(s): NR, RR

Project Title: A Personalized Messaging System for Cancer Screening

SRG Action: Impact/Priority Score: 32
Human Subjects: 30-Human subjects involved - Certified, no SRG concerns
Animal Subjects: 10-No live vertebrate animals involved for competing appl.
Gender: 3A-Only men, scientifically acceptable
Minority: 1A-Minorities and non-minorities, scientifically acceptable
Children: 3A-No children included, scientifically acceptable
Clinical Research - not NIH-defined Phase III Trial

Project Year	Direct Costs Requested	Estimated Total Cost
1	139,671	139,671
TOTAL	139,671	139,671

ADMINISTRATIVE BUDGET NOTE: The budget shown is the requested budget and has not been adjusted to reflect any recommendations made by reviewers. If an award is planned, the costs will be calculated by Institute grants management staff based on the recommendations outlined below in the COMMITTEE BUDGET RECOMMENDATIONS section.

1R43CA162953-01A1 Yuan, Juntao

RESUME AND SUMMARY OF DISCUSSION: This project will develop a web-based personalized messaging system for prostate cancer screening that sends personalized messages (based on history and expressed preferences) via mobile phones. Findings from this study may identify a low cost solution for increasing adherence to cancer screening, thereby reducing cancer related morbidity and mortality. This submission addresses the majority of concerns raised during the previous review with the positive addition of a randomized trial to evaluate the effectiveness of the messaging system. The investigators are a strong team and a good match for the project with considerable expertise. During the discussion the reviewers noted many strengths of the application, notably: a strong approach with a solid theoretical foundation and innovation with respect to conception and design. Some reviewers expressed concern that inadequate detail is provided with regard to measures, analyses and expected results and the integration of current screening recommendations seems to be lacking. Nevertheless, the strengths outweigh the weaknesses of the application and a majority of the review panel concurred that this project would have a high impact on the field.

DESCRIPTION (provided by applicant): Adherence with prostate cancer screening (such as PSA tests, and physician follow-ups), is less than perfect. In large part, this is because members of at-risk populations need to keep track of which tests are required and at what time. As screening guidelines and timetables become more personalized (e.g. different recommendations for follow-up intervals depending on past history of PSA levels), such problems are likely to be exacerbated. Yet, there is a great deal of evidence that early screening and detection are key to better prognoses, lower mortality rates, and lower health-care expenditures. We propose to increase compliance with cancer screening, using prostate cancer as an example, through two-way rich media mobile messaging based on the personalized risk assessment. The product we propose to develop and test will store algorithms required to personalize cancer screening in a central database managed by a rule-based workflow engine, and implemented via messaging on the patient's own mobile phone. Participants will receive a mobile message on the appropriate date stating that it is time for them to schedule and prostate cancer screening appointment with their doctor. Reminder messages will incorporate multimedia, such as a brief video interview with an expert, to reinforce the importance of screening. Participants will be asked to report the results of tests (for example PSA levels), by replying to the message on their mobile phone or by visiting a portal web site. The project will be piloted in a large rural hospital system providing primary and preventive care services, and be evaluated based on a randomized controlled efficacy trial, as well as pre- and post- measures of participant acceptance and engagement. If successful, phase II SBIR funding will be utilized to conduct a longitudinal trial to understand long term efficacy of the messaging intervention, and to develop and deliver messages relevant to screening for other types of cancer.

PUBLIC HEALTH RELEVANCE: We propose to build a technology solution that provides personalized cancer screening reminders via two-way rich media mobile messaging solutions. The solution personalizes each patient's screening schedule based on personal profile and past screening results, and use consumer mobile technology to engage patients. Such interactive reminders could provide a low cost solution for increasing adherence to cancer screening, and help differentiating patients that are at high risk of non-adherence for more aggressive follow-ups.

CRITIQUE 1:

Significance: 1
Investigator(s): 1
Innovation: 1
Approach: 2
Environment: 1

Overall Impact: This revised R43 application aims to develop and test a web-based personalized messaging system for prostate cancer screening reminders. In the Phase I, personalized prostate cancer screening and prevention algorithms and patient education content will be developed from evidence-based prostate screening guidelines. Then, a rule-based workflow engine, a rich messaging system for reminder, and a web-based lightweight patient portal will be developed. Then, the system will be pilot tested in Christus St. Micahel's primary cancer and cancer prevention clinics.

This revised application adequately deals with all the concerns from the previous review. The idea of developing a simple messaging system to remind the prostate cancer screening tests is highly innovative, promising, and feasible. The application deals with a significant public health issue on lack of adherence to recommended cancer screening. The research team has adequate research experience and expertise required to conduct the proposed study. The approach is adequately described with details. Just a few minor concerns on the approach are of concern.

1. Significance:

Strengths

- The public health issue related to lack of adherence to cancer screening is highly significant.
- The significance of personalized cancer screening messaging system is supported by an adequate literature review.
- Evidence to support that messaging will change behaviors has been provided from the literature and will be further explored.

Weaknesses

- None identified.

2. Investigator(s):

Strengths

- The research team includes excellent researchers who have adequate experience and expertise related to the proposed study.
- To address the concern on no investigator with health promotion sciences training, the team now includes Drs. Vandewater, Associate Professor in Health Promotion and Behavior Sciences at the Univ. of Texas, School of Public Health as a consultant.

Weaknesses

- None identified.

3. Innovation:

Strengths

- The idea of developing a messaging system to remind prostate cancer screening tests is highly innovative, promising, and feasible.

Weaknesses

- None identified.

4. Approach:

Strengths

- The research plan is clearly written with adequate information.

- All the concerns on the approach from the previous review have been adequately addressed while adding a randomized trial to test the effectiveness of the messaging system.
- Now, the testing sites include primary care sites.
- More detailed information on the multimedia information that will be sent to the patients is provided.

Weaknesses

- The feasibility of recruiting 200 participants during the timeline (3 months) is not clear.
- More information on psychometric properties of the instruments (TAM and PAM) is needed.
- More details on data management and statistical analysis methods are needed.

5. Environment:

Strengths

- The company has adequate resources and environments to conduct the proposed study.

Weaknesses

- None identified.

Protections for Human Subjects:

Acceptable Risks and Adequate Protections

- Data security measures are in place.

Inclusion of Women, Minorities and Children:

G3A - Only Men, Acceptable

M1A - Minority and Non-minority, Acceptable

C3A - No Children Included, Acceptable

- Because of inherent nature of prostate cancer, only adult men will be included. The ethnic composition will reflect the patient population of Christus St. Micahel's Cancer Center.

Vertebrate Animals:

Not Applicable (No Vertebrate Animals)

Biohazards:

Not Applicable (No Biohazards)

Resubmission:

- This revised application adequately deals with all the concerns from the previous review. Yet, a few minor concerns on the approach are raised from the resubmission.

Resource Sharing Plans:

Not Applicable (No Relevant Resources)

Budget and Period of Support:

Recommend as Requested

- No change recommended.

CRITIQUE 2:

Significance: 2
Investigator(s): 2
Innovation: 2
Approach: 3
Environment: 2

Overall Impact 2: This project will endeavor to increase adherence to cancer screening, in this context prostate cancer, through two-way rich media mobile messaging based on personalized risk assessment. Lack of adherence to cancer screening is a serious problem. The proposal fashions a novel approach to the development and the delivery of reminders using customized two-way rich media mobile messaging. In general, this is a creative, well-conceived and very impressive proposal. The methods and system are potentially extensible to a wide range of clinical problems and behaviors.

1. Significance:

Strengths

- Compliance with cancer screening is known to be rather low and this serves to increase the risks of cancer in a large subset of the population.
- The customized approach to personalized messaging is rather novel in this context and is very promising.
- There is the potential to develop a useful niche product that is both of public health and clinical importance.

Weaknesses

- The commercial potential of such a reminder messaging system is uncertain.

2. Investigator(s):

Strengths

- This is an exceptionally strong multifaceted team with a great breadth and depth of expertise in their respective areas.

Weaknesses

- None noted.

3. Innovation:

Strengths

- This is a rather innovative proposal in conception and in design.

- Two-way rich media mobile messaging is rather novel in the context of healthcare communication and promotion.

Weaknesses

- None noted.

4. Approach:

Strengths

- This is a very well-conceived multifaceted proposal. There is considerable attention to detail.
- Use of evidence-based guidelines is strength.
- A feedback loop to capture user information is important in order to personalize information. This is one of the novel features in this system.
- The use of rich media content in messaging may engage and serve to activate consumers in ways that regular text messages cannot.
- The intervention is strongly theoretically motivated.

Weaknesses

- Prostate cancer screening is a controversial area and this needs to be addressed, at least briefly, in the proposal.
- The generalizability of the results may be limited if sampling is limited to patients who are seen in a cancer center versus a general practitioner's office.
- There are a number of promissory notes in the design and in the execution of the study. But that is largely appropriate for a phase 1.

5. Environment:

Strengths

- Impressive facilities and excellent resources for the purposes of realizing the objectives of this proposal.

Weaknesses

- None noted.

Protections for Human Subjects:

Acceptable Risks and Adequate Protections

- No concerns were identified.

Inclusion of Women, Minorities and Children:

G3A - Only Men, Acceptable

M1A - Minority and Non-minority, Acceptable

C3A - No Children Included, Acceptable

- No concerns indicated.

Vertebrate Animals:

Not Applicable (No Vertebrate Animals)

Biohazards:

Not Applicable (No Biohazards)

Resubmission:

- The authors were highly responsive to the previous critique. As a result, this is a much improved proposal.

Resource Sharing Plans:

Not Applicable (No Relevant Resources)

Budget and Period of Support:

Recommend as Requested

- The proposed budget was appropriate.

CRITIQUE 3:

Significance: 3
Investigator(s): 2
Innovation: 2
Approach: 4
Environment: 4

Overall Impact: Investigators propose to build and validate a personalized cancer screening system, focused initially on prostate cancer. This is a much improved resubmission by an expert team of investigators. It addresses a significant problem in an innovative way, with a solid approach and environment. Evaluation component has been strengthened but remains somewhat weak.

1. Significance:

Strengths

- Lack of adherence to recommended cancer screening is a significant public health problem.
- Some evidence that personalized informative reminders can impact behavior.

Weaknesses

- Increased adherence likely to be moderate / incremental.

2. Investigator(s):

Strengths

- The PI is qualified in expert in mobile technologies and enterprise computing.
- Consultants provide critical expertise in prostate cancer prediction, evaluation, and health care promotion.

- Consultants and site provide critical clinical expertise and recruiting support.

Weaknesses

- Extent of Vickers and Vandewaters involvement is not well specified and may not be sufficient.

3. Innovation:

Strengths

- Moderately innovative combination of technology and expertise.
- Appropriately uses new technology in a way that is still uncommon in health.

Weaknesses

- None noted.

4. Approach:

Strengths

- Workflow engine approach is well suited to goals.
- Plans for lightweight portal and EMR connectivity is s strength.
- Pilot test and evaluation is strength.
- Preventative measures are an aim of system, but no well-recognized preventative measures for prostate cancer exist.

Weaknesses

- More detail is required to assess evaluation – what measures will be used; what analyses will be conducted; expected results and interpretations.

5. Environment:

Strengths

- Pilot site is an excellent environment for engaging with patients and collecting required data.

Weaknesses

- Facilities in support of software development neither indicated but not described.
- Facilities in support of data backup, archiving and disaster recovery neither indicated nor described.
- Facilities in support of server hosting are neither indicated nor described.

Protections for Human Subjects:

Acceptable Risks and Adequate Protections

- Risk to confidentiality adequately addressed by procedural and technical protections.
- Risk of under diagnosis is low as this is a supplement, not a replacement; procedural protections (education) are adequate.

Inclusion of Women, Minorities and Children:

G3A - Only Men, Acceptable

M1A - Minority and Non-minority, Acceptable

C3A - No Children Included, Acceptable

- No concerns were identified.

Vertebrate Animals:

Not Applicable (No Vertebrate Animals)

Biohazards:

Not Applicable (No Biohazards)

Resubmission:

- This is a significantly improved revision, and carefully addresses the weaknesses identified in prior review

Resource Sharing Plans:

Not Applicable (No Relevant Resources)

Budget and Period of Support:

Recommend as Requested

- No concerns were identified.

Additional Comments During Review Discussion:

- In light of the controversy surrounding prostate cancer screening, the choice of this as the clinical domain is problematic. Guideline recommendations differ in this domain. The investigators do not offer a methodology for synthesizing the guideline recommendations from different organizations (e.g., AUA vs USPSTF). As a minor comment, the investigators propose in Aim 1 to address "prostate cancer screening AND PREVENTION algorithms" (emphasis added), although there is no widely agreed or proven strategy for preventing the occurrence of prostate cancer.
- Much of clinical decision support is data-driven. Knowledge bases for CDS require clinical data in order to generate appropriate knowledge interventions for patients or providers. In a standalone system that is proposed, getting those data to the inference engine would be problematic. Though a stub is proposed for linkages to EMRs, the challenge of terminology--how data from different EMRs using different coding schemes are brought together and used to generate knowledge interventions--is not addressed. The investigators do mention HL7 messaging as one way to assist integration and interoperability, but while important this is insufficient. Further, while the use of a popular platform for decision-support programming, such as Drools, would help improve the system, the authors do not address the challenge of knowledge representation and standards for doing this, and this along with the terminology challenge would limit interoperability. This in turn would limit commercialization down the road.

THE FOLLOWING RESUME SECTIONS WERE PREPARED BY THE SCIENTIFIC REVIEW OFFICER TO SUMMARIZE THE OUTCOME OF DISCUSSIONS OF THE REVIEW COMMITTEE ON THE FOLLOWING ISSUES:

PROTECTION OF HUMAN SUBJECTS (Resume): ACCEPTABLE
Potential risks and benefits to human subjects are appropriately outlined; there are no concerns.

INCLUSION OF WOMEN PLAN (Resume): ACCEPTABLE
There are no concerns about the exclusion of women as the proposed study targets adult men who may be at risk for prostate cancer.

INCLUSION OF MINORITIES PLAN (Resume): ACCEPTABLE
There are no concerns about the inclusion of minorities in the proposed studies.

INCLUSION OF CHILDREN PLAN (Resume): ACCEPTABLE
There are no concerns about excluding children in the proposed studies.

COMMITTEE BUDGET RECOMMENDATIONS: The budget was recommended as requested.

NIH has modified its policy regarding the receipt of resubmissions (amended applications). See Guide Notice NOT-OD-10-080 at http://grants.nih.gov/grants/guide/notice-files/NOT-OD-10-080.html.
The impact/priority score is calculated after discussion of an application by averaging the overall scores (1-9) given by all voting reviewers on the committee and multiplying by 10. The criterion scores are submitted prior to the meeting by the individual reviewers assigned to an application, and are not discussed specifically at the review meeting or calculated into the overall impact score. For details on the review process, see http://grants.nih.gov/grants/peer_review_process.htm#scoring.

MEETING ROSTER

Center for Scientific Review Special Emphasis Panel
CENTER FOR SCIENTIFIC REVIEW
Small Business: Healthcare Delivery and Methodologies
ZRG1 HDM-K (10) B
June 23, 2011 - June 24, 2011

CHAIRPERSON

CARTER, LAVERNE MORROW, PHD
MANAGING PRINCIPAL
RESEARCH AND EVALUATION SOLUTIONS, INC.
ALEXANDRIA, VA 22310

MEMBERS

ALTMAN, MICAH , PHD
ASSOCIATE DIRECTOR
HARVARD-MIT DATA CENTER
INSTITUTE FOR QUANTITATIVE SOCIAL SCIENCE
HARVARD UNIVERSITY
CAMBRIDGE, MA 02138

BLUM, TERRY C., PHD
DEAN AND TEDD MUNCHAK PROFESSOR
COLLEGE OF MANAGEMENT
GEORGIA INSTITUTE OF TECHNOLOGY
ATLANTA, GA 30308

FOSTER, KENNETH R., PHD
PROFESSOR
DEPARTMENT OF BIOENGINEERING
UNIVERSITY OF PENNSYLVANIA
PHILADELPHIA, PA 19104

HELD, THOMAS H, MS
PRESIDENT AND CEO
METAMEDIA TRAINING INTERNATIONAL, INC
GERMANTOWN, MD 20874

HICKAM, DAVID HOWARD, MD
PROFESSOR AND ASSOCIATE DIRECTOR
DEPARTMENT OF MEDICINE
PORTLAND VA MEDICAL CENTER
OREGON HEALTH AND SCIENCE UNIVERSITY
PORTLAND, OR 97239

HUNTER, SARAH , PHD
BEHAVIORAL SCIENTIST
DEPARTMENT OF BEHAVIORAL SCIENCES
RAND CORPORATION
SANTA MONICA, CA 90407

IM, EUN-OK , PHD
PROFESSOR
SCHOOL OF NURSING
UNIVERSITY OF TEXAS AT AUSTIN
AUSTIN, TX 78701

JENDERS, ROBERT ALLEN, MD
ASSOCIATE PROFESSOR
DEPARTMENT OF MEDICINE
CEDARS-SINAI MEDICAL CENTER
UNIVERSITY OF CALIFORNIA, LOS ANGELES
LOS ANGELES, CA 900481804

KAUFMAN, DAVID R, PHD
PROFESSOR
DEPARTMENT OF BIOMEDICAL INFORMATICS
COLUMBIA UNIVERSITY
NEW YORK, NY 10032

LEE, LINDA S., PHD
ASSOCIATE DIRECTOR
CLINICAL RESEARCH TRAINING PROGRAM
DUKE UNIVERSITY MEDICAL CENTER
DURHAM, NC 27710

LOPEZ, LENNY , MD
ASSISTANT IN HEALTH POLICY-ASSOCIATE PHYSICIAN
INSTITUTE FOR HEALTH POLICY
MASSACHUSETTS GENERAL HOSPITAL
BRIGHAM AND WOMEN'S HOSPITAL
BOSTON, MA 02114

MACLEOD, BRUCE , PHD
PROFESSOR
DEPARTMENT OF COMPUTER SCIENCE
UNIVERSITY OF SOUTHERN MAINE
PORTLAND, ME 04104

NORMAN, GREGORY JOHN, PHD
ASSOCIATE PROFESSOR
DEPARTMENT OF FAMILY AND
PREVENTIVE MEDICINE
SCHOOL OF MEDICINE
UNIVERSITY OF CALIFORNIA, SAN DIEGO
LA JOLLA, CA 92093

PIENTA, AMY MARIE, PHD
ACQUISITIONS DIRECTOR
INTER-UNIVERSITY CONSORTIUM FOR
POLITICAL AND SOCIAL RESEARCH
UNIVERSITY OF MICHIGAN
ANN ARBOR, MI 48104

RAPCHAK, BARBARA ANN, MS
CEO
LEAP OF FAITH TECHNOLOGIES, INC.
CRYSTAL LAKE, IL 60014

SCHOMMER, JON C, PHD
PROFESSOR AND ASSOCIATE DEPARTMENT HEAD
DEPARTMENT OF PHARMACEUTICAL CARE
AND HEALTH SYSTEMS
SCHOOL OF PHARMACY
UNIVERSITY OF MINNESOTA
MINNEAPOLIS, MN 55455

SMITH, GREGORY L PHD
PROFESSOR
MANAGEMENT DEPARTMENT
KANSAS STATE UNIVERSITY
MANHATTAN, KS 66502

SPITALNICK, JOSH S., PHD
DIRECTOR OF RESEARCH AND CLINICAL SERVICES
VIRTUALLY BETTER, INC
DECATUR, GA 30033

STAMM, BETH HUDNALL, PHD
RESEARCH PROFESSOR AND DIRECTOR
INSTITUTE FOR RURAL HEALTH STUDIES
IDAHO STATE UNIVERSITY
POCATELLO, ID 83209

TACHINARDI, UMBERTO , MD
ASSOCIATE DEAN FOR BIOMEDICAL INFORMATICS
SCHOOL OF MEDICINE AND PUBLIC HEALTH
UNIVERSITY OF WISCONSIN-MADISON
MADISON, WI 53717

WELLS, JENNIFER J., PHD
RESEARCH SCIENTIST
TECHNOLOGICAL ASSISTANCE INSTITUTE
FOR INTELLECTUAL DISABILITY
EUGENE, OR 97401

WHITT-GLOVER, MELICIA C, PHD
PRESIDENT AND CEO
GRAMERCY RESEARCH GROUP
WINSTON-SALEM, NC 27101

WILKIN, NOEL E., PHD
INTERIM ASSOCIATE PROVOST
RESEARCH INSTITUTE OF PHARMACEUTICAL SCIENCES
SCHOOL OF PHARMACY
UNIVERSITY OF MISSISSIPPI
UNIVERSITY, MS 38677

WYRICK, DAVID L, PHD
RESEARCH ASSOCIATE
PREVENTION STRATEGIES, LLC
BROWNS SUMMIT, NC 27214

YANG, DERSHUNG , PHD
PRESIDENT
BRIGHT OUTCOMES, INC.
BUFFALO GROVE, IL 60089

MAIL REVIEWER(S)

DE, SUVRANU , SCD
ASSOCIATE PROFESSOR
DEPARTMENTS OF MECHANICAL, AEROSPACE
AND NUCLEAR ENGINEERING
RENSSELAER POLYTECHNIC INSTITUTE
TROY, NY 12180

KHOURY, DIRAR SHAFIQ, PHD
ASSOCIATE PROFESSOR
BIOMEDICAL ENGINEERING IN MEDICINE
METHODIST HOSPITAL
HOUSTON, TX 77030

MIROTZNIK, MARK STEVEN, PHD
ASSOCIATE PROFESSOR
ELECTRICAL ENGINEERING AND COMPUTER SCIENCE
UNIVERSITY OF DELAWARE
NEWARK, DE 197163130

PANDEY, SUBHASH C, PHD
PROFESSOR AND DIRECTOR
DEPARTMENT OF PSYCHIATRY
NEUROSCIENCE ALCOHOLISM RESEARCH
UNIVERSITY OF ILLINOIS
CHICAGO, IL 60612

SCIENTIFIC REVIEW ADMINISTRATOR

OLUFOKUNBI SAM, DELIA , PHD
SCIENTIFIC REVIEW OFFICER
CENTER FOR SCIENTIFIC REVIEW
NATIONAL INSTITUTES OF HEALTH
BETHESDA, MD 20892

GRANTS TECHNICAL ASSISTANT

VISANA, DIMPLE P, BS
LEAD EXTRAMURAL SUPPORT ASSISTANT
CENTER FOR SCIENTIFIC REVIEW
NATIONAL INSTITUTES OF HEALTH
BETHESDA, MD 20892

Consultants are required to absent themselves from the room during the review of any application if their presence would constitute or appear to constitute a conflict of interest.
