# Supplementary material for: A Personalized Automated Messaging System to Improve Adherence to Prostate Cancer Screening: Research Protocol
Source: JMIR Res Protoc. 2012 Nov 28;1(2):e20. doi: 10.2196/resprot.2398 (PMC3626152; doi:10.2196/resprot.2398)
Supplement: Supplementary file 2 [file resprot_v1i2e20_app2.pdf]

Grant Number: 1R43TR000364-01A1

Principal Investigator(s):
Juntao Michael Yuan

Project Title: A Personalized Messaging System for Cancer Screening

Mr. Ron K. Johnson
General Manager
7020 Covered Bridge Dr.
Austin, TX 787363322

Award e-mailed to: info@ringful.com

Budget Period: 08/18/2012 – 02/17/2013
Project Period: 08/18/2012 – 02/17/2013

Dear Business Official:

The National Institutes of Health hereby awards a grant in the amount of $149,448 (see “Award Calculation” in Section I and “Terms and Conditions” in Section III) to RINGFUL, LLC in support of the above referenced project. This award is pursuant to the authority of 42 USC 241 42 CFR PART 52 15 USC 638 and is subject to the requirements of this statute and regulation and of other referenced, incorporated or attached terms and conditions.

Acceptance of this award including the “Terms and Conditions” is acknowledged by the grantee when funds are drawn down or otherwise obtained from the grant payment system.

Each publication, press release, or other document about research supported by an NIH award must include an acknowledgment of NIH award support and a disclaimer such as “Research reported in this publication was supported by the National Center For Advancing Translational Sciences of the National Institutes of Health under Award Number R43TR000364. The content is solely the responsibility of the authors and does not necessarily represent the official views of the National Institutes of Health.” Prior to issuing a press release concerning the outcome of this research, please notify the NIH awarding IC in advance to allow for coordination.

Award recipients must promote objectivity in research by establishing standards that provide a reasonable expectation that the design, conduct and reporting of research funded under NIH awards will be free from bias resulting from an Investigator’s Financial Conflict of Interest (FCOI), in accordance with 42 CFR Part 50 Subpart F. Subsequent to the compliance date of the 2011 revised FCOI regulation (i.e., on or before August 24, 2012), Awardees must be in compliance with all aspects of the 2011 revised regulation; until then, Awardees must comply with the 1995 regulation. The Institution shall submit all FCOI reports to the NIH through the eRA Commons FCOI Module. The regulation does not apply to Phase I Small Business Innovative Research (SBIR) and Small Business Technology Transfer (STTR) awards. Consult the NIH website http://grants.nih.gov/grants/policy/coi/ for a link to the regulation and additional important information.

If you have any questions about this award, please contact the individual(s) referenced in Section IV.

Sincerely yours,

JEAN RICHELSEN
Grants Management Officer
NATIONAL CENTER FOR ADVANCING TRANSLATIONAL SCIENCES

NIH NGA R | Version: 415 - 06/04/2012 08:01:43 | Generated on: 08/18/2012 00:09:36

Additional information follows

SECTION I – AWARD DATA – 1R43TR000364-01A1

Award Calculation (U.S. Dollars)

**Salaries and Wages**	$60,400
**Fringe Benefits**	$8,446
**Personnel Costs (Subtotal)**	$68,846
**Consultant Services**	$49,000
**Travel Costs**	$9,225
**Other Costs**	$12,600
**Federal Direct Costs**	$139,671
**Approved Budget**	$139,671
**Fee**	$9,777
**Federal Share**	$149,448
**TOTAL FEDERAL AWARD AMOUNT**	$149,448
**AMOUNT OF THIS ACTION (FEDERAL SHARE)**	$149,448

SUMMARY TOTALS FOR ALL YEARS		
**YR**	**THIS AWARD**	**CUMULATIVE TOTALS**
1	$149,448	$149,448

Fiscal Information:

**CFDA Number:**	93.350
**EIN:**	1262973061A1
**Document Number:**	RTR000364A
**Fiscal Year:**	2012

IC	*CAN*	*2012*
TR	8012682	$149,448

NIH Administrative Data:
PCC: CRT50 / **OC**: 414A / **Processed**: RICHELSENJ 08/15/2012

SECTION II – PAYMENT/HOTLINE INFORMATION – 1R43TR000364-01A1

For payment and HHS Office of Inspector General Hotline information, see the NIH Home Page at http://grants.nih.gov/grants/policy/awardconditions.htm

SECTION III – TERMS AND CONDITIONS – 1R43TR000364-01A1

This award is based on the application submitted to, and as approved by, NIH on the above-titled project and is subject to the terms and conditions incorporated either directly or by reference in the following:

a. The grant program legislation and program regulation cited in this Notice of Award.
b. Conditions on activities and expenditure of funds in other statutory requirements, such as those included in appropriations acts.
c. 45 CFR Part 74 or 45 CFR Part 92 as applicable.
d. The NIH Grants Policy Statement, including addenda in effect as of the beginning date of the budget period.
e. This award notice, INCLUDING THE TERMS AND CONDITIONS CITED BELOW.

(See NIH Home Page at ‘http://grants.nih.gov/grants/policy/awardconditions.htm’ for certain references cited above.)

Carry over of an unobligated balance into the next budget period requires Grants Management Officer prior approval.

This award is subject to the requirements of 2 CFR Part 25 for institutions to receive a Dun & Bradstreet Universal Numbering System (DUNS) number and maintain an active registration in

the Central Contractor Registration. Should a consortium/subaward be issued under this award, a DUNS requirement must be included. See http://grants.nih.gov/grants/policy/awardconditions.htm for the full NIH award term implementing this requirement and other additional information.

Based on the project period start date of this project, this award is likely subject to the Transparency Act subaward and executive compensation reporting requirement of 2 CFR Part 170. There are conditions that may exclude this award; see http://grants.nih.gov/grants/policy/awardconditions.htm for additional award applicability information.

In accordance with P.L. 110-161, compliance with the NIH Public Access Policy is now mandatory. For more information, see NOT-OD-08-033 and the Public Access website: http://publicaccess.nih.gov/.

This award represents the final year of the competitive segment for this grant. Therefore, see the NIH Grants Policy Statement Section 8.6 Closeout for closeout requirements at: http://grants.nih.gov/grants/policy/#gps .

A final Federal Financial Report (FFR) (SF 425) must be submitted through the eRA Commons (Commons) within 90 days of the expiration date; see the NIH Grants Policy Statement Section 8.6.1 Financial Reports, http://grants.nih.gov/grants/policy/#gps, for additional information on this submission requirement. The final FFR must indicate the exact balance of unobligated funds and may not reflect any unliquidated obligations. There must be no discrepancies between the final FFR expenditure data and the Payment Management System's (PMS) cash transaction data.

A Final Invention Statement and Certification form (HHS 568), (not applicable to training, construction, conference or cancer education grants) must be submitted through the eRA Commons (Commons) within 90 days of the expiration date.

Furthermore, unless an application for competitive renewal is submitted, a final progress report must also be submitted within 90 days of the expiration date. Institute/Centers may accept the progress report contained in competitive renewal (type 2) in lieu of a separate final progress report. Contact the awarding IC for IC-specific policy regarding acceptance of a progress report contained in a competitive renewal application in lieu of a separate final progress report.

NIH strongly encourages electronic submission of the final progress report and the final invention statement through the Closeout feature in the Commons. If the final progress report and final invention statement are not submitted through the Commons, a copy can be emailed or sent to the contacts listed below. Copies of the HHS 568 form may be downloaded at: http://grants.nih.gov/grants/forms.htm.

Submissions of the final progress report and HHS 568 may be e-mailed as PDF attachments to the NIH Central Closeout Center at: DeasCentralized@od.nih.gov.

Paper submissions of the final progress report and the HHS 568 may be faxed to the NIH Central Closeout Center at 301-480-2304 or mailed to the NIH Central Closeout Center at the following address:

NIH/OD/OER/DEAS
Central Closeout Center
6705 Rockledge Drive, Room 2207
Bethesda, MD 20892-7987 (for regular or U.S. Postal Service Express mail)
Bethesda, MD 20817 (for other courier/express mail delivery only)

The final progress report should include, at a minimum, a summary of progress toward the achievement of the originally stated aims, a list of significant results (positive and/or negative), a list of publications and the grant number. If human subjects were included in the research, the final progress report should also address the following:

Report on the inclusion of gender and minority study subjects (using the gender and minority Inclusion Enrollment Form as provided in the PHS 2590 and available at http://grants.nih.gov/grants/forms.htm).

Where appropriate, indicate whether children were involved in the study or how the study was relevant for conditions affecting children (see NIH Grants Policy Statement Section 4.1.15.7 Inclusion of Children as Subjects in Clinical Research at URL http://grants.nih.gov/grants/policy/#gps).

Describe any data, research materials (such as cell lines, DNA probes, animal models), protocols, software, or other information resulting from the research that is available to be shared with other investigators and how it may be accessed.

Any other specific requirements set forth in the terms and conditions of the award must also be addressed in the final progress report.

Note, if this is the final year of a competitive segment due to the transfer of the grant to another institution, then not all the requirements stated above are applicable. Specifically a Final Progress Report is not required. However, a final FFR is required and should be submitted electronically as noted above. In addition, if not already submitted, the Final Invention Statement is required and should be sent directly the assigned Grants Management Specialist.

Treatment of Program Income:
Additional Costs

SECTION IV – TR Special Terms and Conditions – 1R43TR000364-01A1

RESTRICTION: The present award is being made without a currently valid certification of IRB approval for this project with the following restriction: Only activities that are clearly severable and independent from activities that involve human subjects) may be conducted pending the NCATS? acceptance of the certification of IRB approval. The certification of IRB approval must be submitted to NCATS. No funds may be drawn down from the payment system and no obligations may be made against Federal funds for research involving human subjects at any site engaged in such research for any period not covered by an Office for Human Research Protections Assurance and an IRB approval consistent with the requirements of 45 CFR Part 46. Failure to submit the certification of IRB approval to the NCATS or to otherwise comply with the above requirements can result in suspension and/or termination of this award, withholding of support, audit disallowances, and/or other appropriate action.

See the NIH Grants Policy Statement, October, 2011, (http://grants2.nih.gov/grants/policy/nihgps_2011/index.htm), for specific requirements related to the protection of human subjects, which are applicable to and a term and condition of this award.

This award is subject to the conditions set forth in PA-11-096, "PHS 2011-02 Omnibus Solicitation of the NIH, CDC, FDA and ACF for Small Business Innovation Research Grant Applications (Parent SBIR [R43/R44])," which are hereby incorporated by reference as special terms and conditions of this award. Copies of this Funding Opportunity Announcement can be found at the following link: http://grants.nih.gov/grants/guide/pa-files/pa-11-096.html.

This award is issued in accordance with the NIH fiscal policies described in NIH Guide Notice OD-12-036.

This award transfers administrative responsibility of this grant from the National Center for Research Resources (NCRR) to the National Center for Advancing Translational Sciences (NCATS).

This award reflects the NCATS' acceptance of the certification that all key personnel have completed education on the protection of human subjects, in accordance with NIH policy, "Required Education in the Protection of Human Research Participants" (http://grants.nih.gov/grants/guide/notice-files/not-od-01-061.html).

Any individual involved in the design and conduct of the study that is not included in the certification must satisfy this requirement prior to participating in the project. Failure to comply

can result in the suspension and/or termination of this award, withholding of support of the continuation award, audit disallowances, and/or other appropriate action.

Intellectual property rights: Normally the grantee organization retains the principal worldwide patent rights to any invention developed with United States Government support. Under Title 37 Code of Federal Regulations Part 401, the Government receives a royalty-free license for its use, reserves the right to require the patent holder to license others in certain circumstances, and requires that anyone exclusively licensed to sell the invention in the United States must normally manufacture it substantially in the United States.

Rights and obligations related to inventions created or reduced to practice as a result of this award are detailed in 35 U.S.C. 205 and 37 CFR Part 401. These inventions must be reported to the Extramural Invention Reporting and Technology Resources Branch, OPERA, NIH, 6705 Rockledge Drive, MSC 7980, Bethesda, MD 20892-7080, (301) 435-1986. For additional information, access the NIH link on the Interagency Edison web site (www.iedison.gov) which includes an electronic invention reporting system, reference information and the text to 37 CFR 401.

To the extent authorized by 35 U.S.C., Section 205, the Government will not make public any information disclosing an NIH-supported invention for a 4-year period to allow the grantee organization a reasonable time to file a patent application, nor will the Government release any information that is part of that patent application.

The fee provided as part of this Notice of Grant Award is in addition to direct and facilities and administrative costs. The fee is to be drawn down from the HHS Payment Management System in increments proportionate to the drawdown of costs.

Allowable costs conducted by for-profit organizations will be determined by apply the cost principles of Contracts with Commercial Organizations set forth in 48 CFR, Subpart 31.2.

In addition to the PI, the following individuals are named as key personnel:

Norman Richards
Andrew Vickers
George Finley
Elizabeth Vandewater
Ju Long

Written prior approval is required if any of the individual(s) named above withdraws from the project entirely, is absent from the project during any continuous period of 3 months or more, or reduces time devoted to the project by 25 percent or more from the level that was approved at the time of award.

If the grantee plans to issue a press release concerning the outcome of NCATS grant-supported research, it should notify the NCATS Office of Communications at 301-435-0888 in advance to allow for coordination.

The NCATS WWW home page is at http://ncats.nih.gov/

STAFF CONTACTS

The Grants Management Specialist is responsible for the negotiation, award and administration of this project and for interpretation of Grants Administration policies and provisions. The Program Official is responsible for the scientific, programmatic and technical aspects of this project. These individuals work together in overall project administration. Prior approval requests (signed by an Authorized Organizational Representative) should be submitted in writing to the Grants Management Specialist. Requests may be made via e-mail.

Grants Management Specialist: Leslie Le
Email: leleslie@mail.nih.gov **Phone**: (301) 435-0856 **Fax**: (301) 480-3777

Program Official: Rosemarie Filart
Email: filartr@mail.nih.gov **Phone**: 301.435.0178 **Fax**: 301.480.3661

SPREADSHEET SUMMARY
GRANT NUMBER: 1R43TR000364-01A1

INSTITUTION: RINGFUL, LLC

Budget	***Year 1***
Salaries and Wages	$60,400
Fringe Benefits	$8,446
Personnel Costs (Subtotal)	$68,846
Consultant Services	$49,000
Travel Costs	$9,225
Other Costs	$12,600
FEE	$9,777
TOTAL FEDERAL DC	$139,671
TOTAL FEDERAL F&A	
TOTAL COST	$149,448
