# Supplementary material for: A Personalized Automated Messaging System to Improve Adherence to Prostate Cancer Screening: Research Protocol
Source: JMIR Res Protoc. 2012 Nov 28;1(2):e20. doi: 10.2196/resprot.2398 (PMC3626152; doi:10.2196/resprot.2398)
Supplement: Supplementary file 3 [file resprot_v1i2e20_app3.pdf]

CHRISTUS Health
*Clinical Excellence Services*
*Institutional Review Board*

**CHRISTUS Health Institutional Review Board**

Date: August 22, 2012

To: Michael Yuan, MD

Re: **Protocol # 2012-026** — A Personalized Messaging System to Improve Cancer Screening

Approval Status: Minor Modifications Required

Dear Dr. Yuan:

This letter is to inform you that CHRISTUS Health IRB has reviewed the above research study and requires minor changes. The research protocol is not approved until documents are submitted indicating the following conditions have been met:

- Clarify in the Informed Consent document how Electronic Medical Records (EMR) might be accessed, who will access them, why the full EMR needs to be accessed and the encryption process
- Submit educational material, and all forms of patient communication to the IRB for vetting

Once the documentation is submitted and reviewed, a letter of IRB Approval of this study will be issued. Until full approval is received in the form of a letter from the IRB, no subjects may be contacted, consented, or enrolled in the study and the study may not be initiated. Research conducted without final IRB approval is a violation of federal regulations and institutional policies.

If you have any questions, please feel free to contact the IRB at 281-936-3593.

Sincerely,

Darrell Dixon, MD
CHRISTUS Health IRB Chair

Cc: Michael Finley, MD

**CHRISTUS Health Research Office**
**2707 North Loop West**
**Houston, TX 77008**
**281-936-3588**
